# The economic imperatives for technology enabled wellness centered healthcare

**DOI:** 10.1057/s41271-022-00356-8

**Published:** 2022-08-03

**Authors:** Graham B. Jones, Justin M. Wright

**Affiliations:** 1 Connected Health Program, Global Drug Development, Novartis Pharmaceuticals, 1 Health Plaza, East Hanover, NJ 07936 USA; 2 Clinical and Translational Science Institute, Tufts University Medical Center, 800 Washington Street, Boston, MA 02111 USA

**Keywords:** Wellness, Telemedicine, Digital biomarkers, Digital health technologies, P4 medicine

## Abstract

A 2020 World Health Organization report underscored the impact of rising healthcare spending globally and questioned the long-term economic sustainability of current funding models. Increases in costs associated with care of late-stage irreversible diseases and the increasing prevalence of debilitating neurodegenerative disorders, coupled with increases in life expectancy are likely to overload the healthcare systems in many nations within the next decade if not addressed. One option for sustainability of the healthcare system is a change in emphasis from illness to wellness centered care. An attractive model is the P4 (Predictive, Preventative, Personalized and Participatory) medicine approach. Recent advances in connected health technology can help accelerate this transition; they offer prediction, diagnosis, and monitoring of health-related parameters. We explain how to integrate such technologies with conventional approaches and guide public health policy toward wellness-based care models and strategies to relieve the escalating economic burdens of managed care.

## Key messages

- New digital technologies offer unprecedented opportunities for prediction, diagnosis, monitoring, and coaching to improve people’s health.
- Use of digital health technologies by both patients and providers has expanded dramatically through the COVID-19 pandemic and these technologies are poised to become mainstream components of managed care.
- A major opportunity exists to deploy digital technologies in preventative medicine with the potential to relieve major economic burden on managed healthcare by delaying or preventing disease onset.

## Introduction

**Fig. 1:**
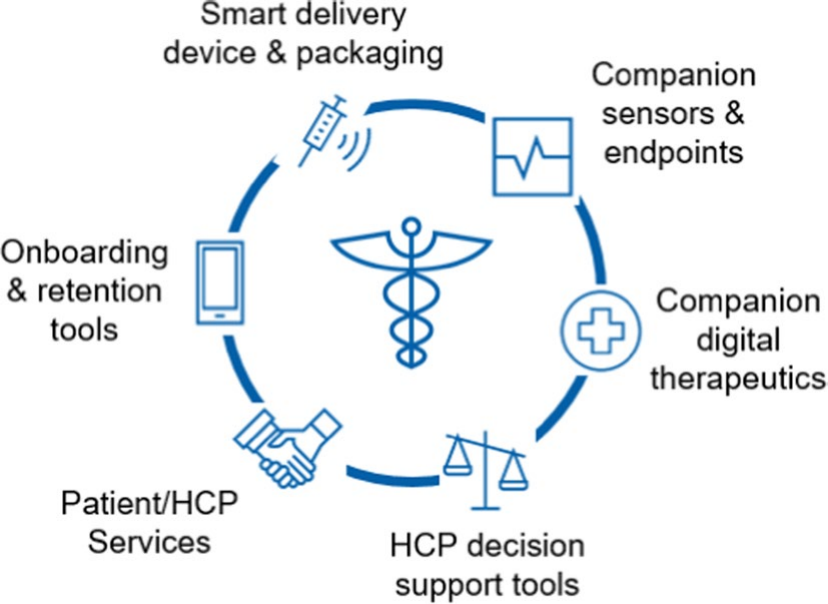
Components of contemporary connected health ecosystems

A recent report from the World Health Organization documents spiraling costs of healthcare during the period 2000–2018. Expenditures reached over $8 trillion—equivalent to 10% of global Gross Domestic Product (GDP) [1]. In the United States (US), costs continue to rise both in real terms and as a proportion of GDP, a major component of which is directed towards the treatment of late stage, typically irreversible forms of cancer, heart disease, and neurological disorders [2]. Despite prevalence of such diseases, average life expectancy has been rising globally for some time, meaning that the financial and logistic burdens on healthcare systems will increase accordingly [3]. Alzheimer’s and Parkinson’s diseases alone represent a potentially insurmountable obstacle for current healthcare systems as patients require labor intensive assisted care focused on quality of life [4]. Thus, many managed healthcare systems (where patients are treated by general and specialist medical practitioners through insurance funded provider networks) may become unsustainable and require intervention and a shift of emphasis to remain financially viable long term. Provision of healthcare by sovereign nations typically involves interaction with healthcare providers (HCP’s), health regulatory authorities, manufacturers (drugs, devices, etc.) and funders (insurers, federal funding bodies, and individuals). Guidance on medical best practice varies and policy changes tend to occur in incremental steps, unless precipitated by landmark events—examples include availability of new vaccines, low-cost genetic sequencing methods, and technology for the introduction of fluoride into drinking water. We believe that collectively, the field of Connected Health now presents such an event and offers an opportunity to reshape healthcare policy. Connected Health (sometimes referred to as e-Health, m-Health, Digital Health) represents a philosophy whereby health related data captured through electronic devices are shared electronically among patients, Healthcare Providers (HCP’s), and funders to improve outcomes (Fig. 1) [5].

Connected Health involves ‘smart’ packaging, for example, a patient or caregiver records a drug dose, then a ‘smart’ electronic device guides a patient to optimal health behaviors, or a sensor alerts an HCP or patient to the potential of an adverse event. Sharing these data securely among health providers and patients will enhance medical decision making to a degree of health maintenance previously unimaginable. Advances in computing power and miniaturization, our understanding of disease etiology, and availability of ever more accurate biosensors are transforming the field; already they have markedly and positively influenced treatment of diabetes, obesity, and cardiovascular disease [6]. Adoption of Connected Health approaches proceeds at a moderate pace despite the revolutionary promise such disruptive technologies could offer. Adoption involves an interface of the high technology and biopharmaceutical industries whose rates of innovation and degree of regulatory oversight are very different. And, although there has been near explosive growth of health-related consumer products globally (fitness trackers, smart watches, etc.) emergence of devices with clinically validated digital endpoints lags [7]. These are required for regulatory approval in most countries and may also prompt physician endorsement and end payer (funder) reimbursement approval, given the need for standardized and verified outcome measures [8]. While progress on this front continues, the COVID pandemic has had a positive impact on the development of Connected Health options. HCP’s have eagerly adopted telemedicine approaches to provide managed care and look likely to retain many aspects of this post-pandemic [9]. Notably in the US market (where insurance providers determine access to care through networks) most major insurers introduced provisions for telemedical interventions to become *reimbursable*, provisions likely to be preserved post-pandemic, in part based on economic benefits and efficiencies in patient flow [10]. Data privacy laws and regulations abound, and it will take motivated populations globally to embrace capture of such sensitive health data and their accompanying transmission, storage, and retrieval technologies. Widespread deployment of tracking apps during the pandemic, however, re-illustrates the power of population level data to identify and monitor disease dynamics.

Below we outline how connected health technologies could have broad impact on patient healthcare by complementing traditional diagnostic, screening, and monitoring methods. We offer the case for these technologies to spearhead wellness-centric care models and sustained impact on healthcare economics. Fully capitalizing on this major opportunity will require active discussion among public health policy-makers, regulatory bodies, insurance companies, healthcare providers, and patient groups. We suggest implementation models.

## Connected Health in action

**Fig. 2:**
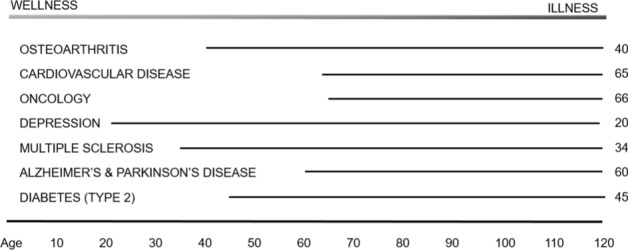
Average age on onset of some common medical conditions globally [13]

Deployment of Connected Health systems to enhance patient healthcare requires an objective look at the status quo. In most nations, patient healthcare focuses on the treatment of *disease* through detection, diagnosis, staging, intervention, and management (surgical, therapy, lifestyle modifications, etc.). This focus on managing and treating illness typically comes at the expense of approaching healthcare from the perspective of *wellness* and prevention. In the US healthcare system, for example, the industry guides physicians in networks using approved procedural codes (such as the CPT and HCPCS) which determine which actions (diagnostics, therapeutics) can be billed and reimbursed, and defines or limits the scope of care [11]. And in many countries where health insurance is used by patients to offset medical costs, actuarial analysis informs setting of rates for insurance premiums by calculating lifetime risk factors with a high degree of precision [12]. Figure 2 illustrates the average age of disease onset in seven therapeutic categories based on global data [13].

**Fig. 3:**
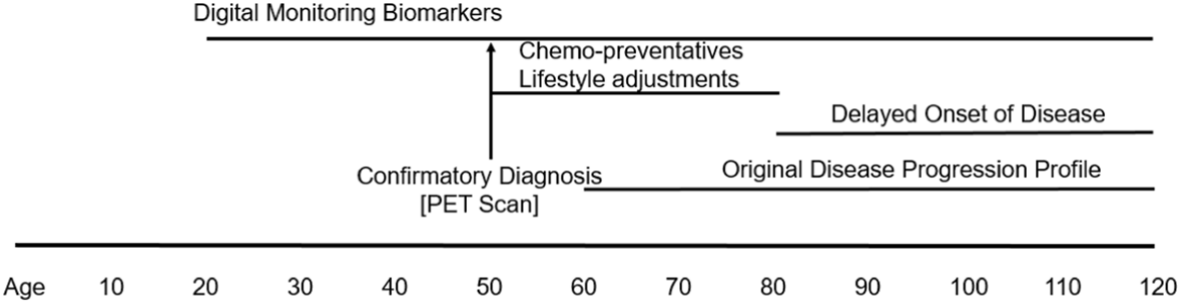
How connected health early detection methods might be applied to neurodegenerative disease care

**Fig. 4:**
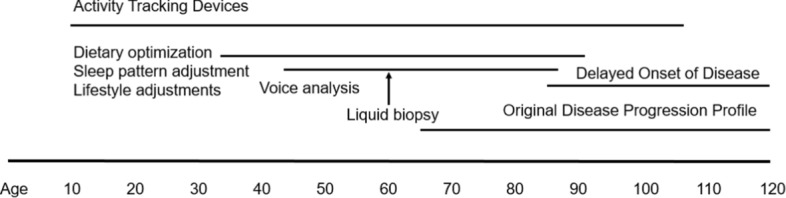
How connected health early detection methods might be applied to cardiovascular disease care

When factored alongside the theoretical limit on life expectancy of 115 years [14], the financial burden of the diseases becomes glaringly apparent, and the need to shift emphasis from illness to wellness compelling. Take the example of treatment of cognitive disorders such as Alzheimer’s disease. Delaying onset of disease by even a few years could result in considerable benefit by reducing strain on health providers globally (Fig. 3). Societies might achieve these savings through early detection, by deploying Connected Health monitoring technologies to high-risk individuals (genomics, familial history, for example) to plot changes in key digital biomarkers over time, such as gait analysis [15], speech aberrations [16], and motor functional precision [17]. Many of these sensors are now available on consumer devices (smart watches). As cost and prices of such goods decrease, they will become widely available globally. The purpose of these ‘early warning’ measures would be to alert a patient to the need for a confirmatory assessment and diagnosis through an HCP. The current gold standard for such diagnosis is a Positron Emission Tomography (PET) scan [18], although liquid biopsies (such as serum neurofilament light chain [sNfL]) could become near term alternatives [19]. If the imaging were to confirm presence of plaques signifying the early onset, or ‘pro-dromal’ diseased state, then researchers, HCPs, and patients could initiate specific interventions to delay the onset of the full-blown disease. Examples include clinical trials of a plaque-preventing drug and associated lifestyle changes such as a high-antioxidant diet, cognitive behavioral therapy, and enhanced physical activities [20–23]. In addition to reducing disease burden, this approach would also help identify potentially large cohorts of patients with whom researchers can conduct clinical trials (where sufficient scale, study power, and diversity is critical) to help the industry realize as-yet elusive disease modifying agents [24]. A similar interventional approach could be applied to the treatment of cardiometabolic and cardiovascular disorders. Tian and Meng demonstrated that diet, exercise, sleep quality, and lifestyle factors all play roles in these conditions (Fig. 4) [25]. Even rudimentary consumer products widely available in Europe, China, and the US now measure Heart-Rate Variability (HRV) as a function of strain and recovery rate. A patient tracking HRV from teenage years onward (thereby establishing good baseline metrics) might be persuaded, over time, to optimize diet and sleep cycles to sustain and even enhance scores for this biomarker [26]. Patients could supplement data they capture from tracking with data from residential ‘smart’ devices. Such tools, including voice assistants, make up the ‘Internet of Medical Things’ (IoMT). Collectively they form a composite biomarker to signal a defined risk factor such as dramatic spikes in respiratory rate tied to HRV changes. This event might indicate benefit of a subsequent action to confirm a diagnosis with the patients HCP (for example, submission of a liquid biopsy sample or performing an ECG) to allow an HCP to prescribe specific adjustments for managing the patients’ health. The over-arching objective would be to delay onset of disease and encourage the patient to become an active participant in efforts to maintain better health. Such a mindset shift would have considerable consequence in the US, where reduction in the burden of treatment of cardiovascular related diseases would allow redirection of resources to myriad areas of unmet need.

The combination of Connected Health systems and digital monitoring biomarkers could have a major impact on health econometrics, but are unlikely to do so in isolation. Given the vast wealth of biomarkers in accessible biological fluids (blood, sweat, tears, saliva, breath) [27], coupled with advances in molecular detection technologies (such as genetic sequencing, protein mass spectrometry, high performance chromatography) [28], a holistic health management program could emerge. Already, several companies offer mail-in biopsy services to consumers. Some analyze metabolites (the dip.io, TestCard) and genetic markers (like 23 & Me) [29]. Others provide services at specialized diagnostics facilities (including mobile centers for HIV or COVID testing). Another option could be through community-based commercial facilities. Retail pharmacy chains across countries offer considerable potential as many have developed large networks through acquisition (including CVS (US), JD Pharmacy (China), Apollo (India), Goodlife (East Africa). Some of these pharmacies offer on-demand health clinic services, such as the ‘5 min clinic’ offered by CVS for basic diagnostic assessment. Many have served as testing and vaccination points for the COVID pandemic. Given that some patients may find a retail pharmacy nearby rather than a hospital, could these establishments become community-based hubs where patients deposit or submit diagnostic samples with results later communicated to the patient (and their HCP) via a device or cloud based service? In countries where retail pharmacies are limited, another possibility could be community-based grocery stores. In the US, the retail giant Wal Mart and supermarket chain Kroger both offer customers access to Higi smart healthcare stations which analyze basic cardiovascular information (BMI, pulse, blood pressure) and provide guidance on wellness [30]. Such capabilities could be helpful in densely populated regions around the world where patients endure long wait times to see an HCP. China has taken a lead in such virtual clinic approaches through the Ping An Good Doctor (PAGD) network that offers a remote ‘1 min clinic’ service powered by AI [31]. New COVID-inspired diagnostic and tracking technologies may have opened more widely a door for patients to accept such services, particularly if they gain a degree of control over sharing of information—with whom and by what means [32].

There are many obstacles for wide scale deployment of Connected Health approaches at the population level, including financial, logistic, data privacy, and ethical considerations. COVID induced digital experiences (testing) and rapid advances in smart devices (the IoMT), data architectures (including the new internet protocol known as IPv6 which vastly increases the total number of available addresses) [33], advanced cellular and other networks (5G and low-power wide-area networks) [34], and new interoperability standards for secure transfer of health data such as the Fast Healthcare Interoperability Resources (FHIR) [35] suggest the time is right for this paradigm shift towards sustainability of managed healthcare.

## The emerging role of digital sensor technologies in healthcare

It is clear that healthcare systems globally are under increasing financial pressure, and many will become unsustainable economically [2]. In the US alone, increases in medical costs coupled with increased life expectancy will render state provided care (the Medicare and Medicaid system for retirees) untenable within the next decade if not addressed (Table 1). Principal components of this looming healthcare cost tsunami are neurodegenerative diseases including Alzheimer’s and Parkinson’s diseases which account for substantial impact on Disability Adjusted Life Years (DALY) and require specialized care [36]. Even raising retirement age to 75 may not address this issue in many nations. Thus, societies must rethink how they will administer and fund healthcare at national, state, neighborhood, and individual levels. An early advocate for such a paradigm shift was Leroy Hood who introduced the P4 medicine approach: Predictive, Preventative, Personalized and Participatory medicine [37]. The approach takes into account current administrative and financial incentives in healthcare practice. For example, in countries where insurance companies reimburse HCP’s for patient care, few if any incentives pertain to patient wellness. Patient care tends to be episodic when patients only interacting with HCP’s if they are sick. With the exception of tobacco product use, insurance companies link premiums paid by patients to age groups, aggregated across the population and independent of habits, history, and pre-existing conditions [38]. Thus, labor-intensive care for patients diagnosed with late-stage terminal illnesses accounts for much of the spiraling in costs of healthcare [39]. A different approach might have identified these problems much earlier in a patients’ lifespans [2]. Those we describe in this Viewpoint, if deployed at scale, could help turn the healthcare system towards sustainability (Table 1). Given the annualized costs of treatment for the eight diseases listed, delaying onset by merely one calendar year would translate to savings in the hundreds of billions of dollars in the US alone.

**Table 1:** Annualized economic cost of treating different indications

Indication	US cost ($B)	US cases (M)	Global cases (M)	References
Osteoarthritis	186	33	300	[40]
Cardiovascular	320	122	523	[41]
Oncology	150	1.9^b^	17^b^	[42]
Depression	210	17	264	[43]
Multiple Sclerosis	28	0.4	2.1	[44]
Alzheimer’s^a^	305	5.8	50	[45]
Parkinson’s^a^	52	1	10	[46]
Type 2 Diabetes	245	31	462	[47]
Total	$1.5 T	212 M	1628 M	

Data as of 2/2021

^a^Represents direct + indirect costs

^b^New cases annually

**Fig. 5:**
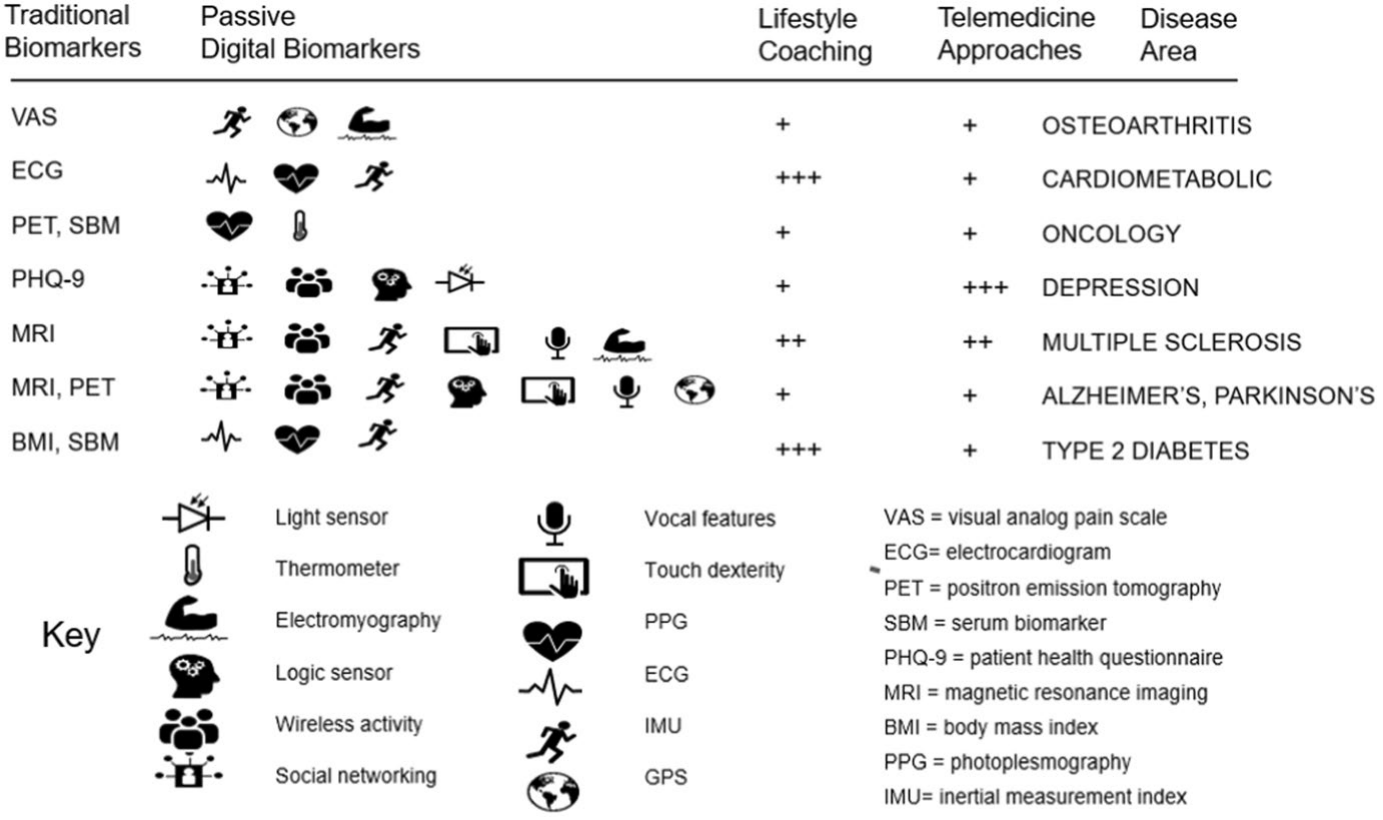
How digital sensors can complement traditional measures for disease management

This will not, however, be a simple process. Savings derived from early diagnosis (Figs. 2 and 3) would need to be re-invested in preventative health services, and health insurance premiums tied to positive patient behaviors. There is some movement towards this now, with premiums lowered for those who do not smoke and discounts for health club memberships. Lessons from adjacent industries are also relevant, such as safe driver automobile insurance premium discounts offered to customers who consent to placement of motion sensors in their vehicles [48]. Coupling exercise-induced heart rate variability (easily tracked by wrist worn monitors) to lowered premiums for those at risk of cardiometabolic disease could become a viable option and is the basis for several successful healthcare businesses that provide active coaching to patients [49–51]. Ethically, removing high-risk individuals from the general health insurance pool (as is the case in automobile insurance for repeat drink-driver offenders) is unpalatable but some effort to reward positive patient behaviors could balance pecuniary consequences for destructive behaviors. The myriad devices, sensors, and systems available to coach individuals towards good health and sustain wellness into late life are now relatively affordable. Equally importantly, they facilitate integration of data to produce composite metrics for each person [52]. Just as an automobile tachometer or temperature gauge alerts us to prevent engine damage, so might a wrist-worn blood pressure sensor for our cardiovascular system prevent organ or tissue damage. For those suffering from early onset neuro-motor disorders, a gait analyzer in a smartphone might provide a warning alert to guide walking as a lane change sensor warning does in an automobile. Other devices and systems present in the home could supplement on-body sensors. These include smart scales, smart mirrors, smart refrigerators, and ever-improving voice assistants. Consumers can also buy diagnostic kits, including some which provide at home readouts of metabolites and complex biomarkers associated with disease, including urinalysis for kidney disease and cystitis [53, 54]. Popularity of these is likely to grow globally, as has familiarity and acceptability with COVID testing. To standardize and incentivize development of new forms of biomarkers and digital measures, the US Food and Drug Administration and National Institutes of Health issued definitive guidelines through the Biomarkers and Endpoints (BEST) initiative. The biopharmaceutical and health industries will need to help define reliable endpoints (both traditional and digital) to establish outcome measures for wellness [55]. Figure 5 presents traditional and digital biomarkers relevant for major disease categories. Availability of consumer products with sensors providing valuable health-related read-outs is increasing, and over time we expect clinical validation of these products for certain medical applications. For example, in cardiovascular disease management, along with the ‘gold standard’ electrocardiogram, devices such as the Apple Watch are capable of providing ECG like data in addition to heart rate and blood chemistry readouts (photoplesmography) and activity data (from IMU sensors). Together these provide a composite data-stream for diagnostic event monitoring and for encouraging healthy lifestyle habits by the patient, with the assistance of health ‘coaches’ when useful [49–51]. For neurodegenerative diseases, Industry has produced a multitude of sensors in consumer devices to track eye movement, acoustics, dexterity, motion, balance, and cognitive ability. Given the prolonged pre-onset stages of diseases such as Alzheimer’s and Parkinson’s, it is important to pursue early diagnosis, followed by monitoring through later stages. The gold standards for disease confirmation (currently MRI and PET imaging) suffer from being costly and inaccessible to many across the globe. Even though the design of such consumer technologies allows for largely passive capture of data, there may be limitations on their usefulness in later stage care in neurodegenerative diseases. In these situations, the role of caregivers becomes essential, be they family members, home care assistants, or healthcare providers in specialized residential facilities. Many countries have established ‘assisted living’ communities that provide housing in facilities that offer on-site specialized care for retirees and patients suffering from a variety of ailments. Increasingly these communities have wireless networks and in-room alert sensors and may be ideal venues for deployment of Connected Health sensors (Fig. 5) as part of patient care.

## Next steps

**Fig. 6:**
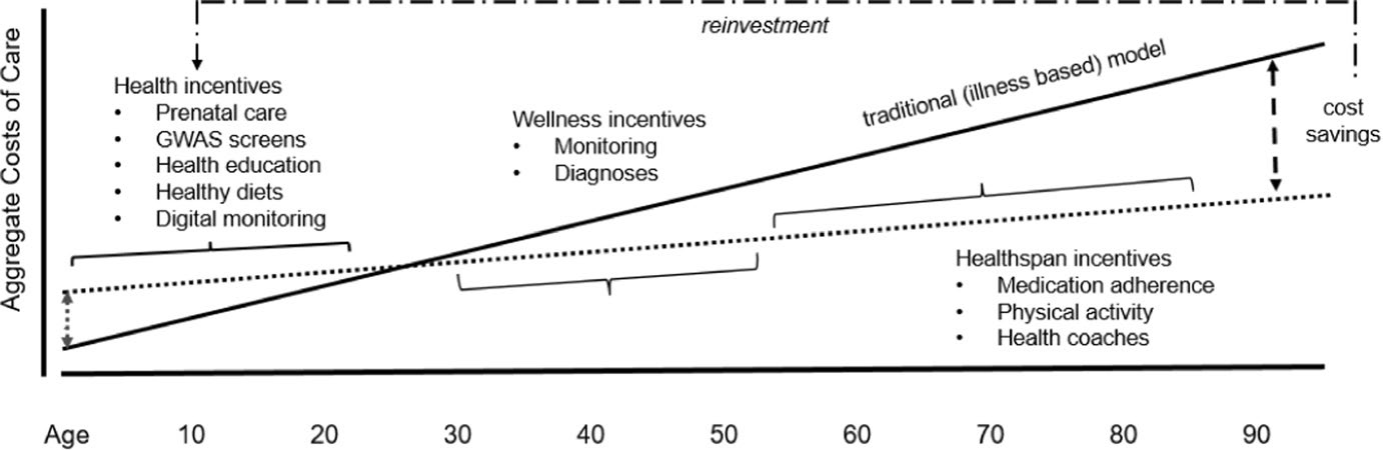
Redeployment of capital savings in a sustainable wellness based healthcare ecosystem

Design, support, and oversight of sustainable healthcare systems is in the interest of every sovereign nation. Success in achieving this goal will require overcoming numerous challenges. Countries will need new financial models for healthcare, new public–private-partnerships to foster patient-centric innovation, and regulatory environments that stimulate investment in healthcare. We already see warning signs about financial instability of current models, including the burden of treating late-stage diseases in populations with increased life expectancy and the impact of neurodegenerative disorders [1, 2]. Shifting the focus of healthcare and its finance to wellness from illness represents a refreshing and potentially sustainable approach to this looming crisis. Achieving this turnaround will require patients, healthcare providers, and funding bodies (federal and state or insurance companies) to appreciate and embrace the tenets of P4 medicine. High-performance digital diagnostic and monitoring tools can play a key role. During the pandemic many patients and HCP’s experienced benefits of telemedicine and connected health devices. Technological advances in sensor and device design can only enhance their adoption post-pandemic. Collectively, these approaches pave a path toward wellness centric healthcare. If deployed successfully, we imagine an inflexion point in terms of net cost savings as depicted in Fig. 6. We also imagine:

- Wellness incentives to encourage early-stage diagnosis and monitoring would lower overall expenditures on chronic, irreversible diseases until much later in a person’s lifespan.
- In the interim, incentives to delay onset of these diseases (for coaching, physical activity, medication) would help keep people from requiring chronic care.
- Financial savings accrued by the care providers (state, federal, or insurance based) would be deployed towards early-stage health incentives including pre-natal services, genomic, epigenetic, and proteomic screens at early age.
- Wellness incentives to encourage early-stage diagnosis and monitoring would lower overall expenditures on chronic, irreversible diseases until much later in a person’s lifespan.
- Customized health plans (for example, diet, allergy factors) would follow, with personalized education programs (Fig. 6).
- One could imagine patients in ‘Generation Z’ (defined as those reaching adulthood in the second decade of the twenty-first century) to be avid adopters of health and sensor technology. Their use would establish a baseline for longitudinal monitoring and outcome measures to inform future generations.

## Conclusions

Recent advances in digital health technologies offer major opportunities for use in prediction, diagnosis, monitoring, and encouraging individuals to participate in improving their health, or at least delaying need for labor-intensive chronic care. Buoyed by their use during the COVID pandemic, telemedical approaches will become cornerstones of managed healthcare. Then, by tracking health related parameters across the wellness–illness continuum we can integrate these data with conventional measures of health, and in doing so to help guide health policy. Wellness-based care models and strategies can help relieve the escalating economic burden of managed care by delaying or preventing onset of debilitating, irreversible neurodegenerative and cardiometabolic diseases. Concerted discussion among funding bodies, regulatory agencies, healthcare providers, and patient groups will be essential, as will meaningful debates with healthcare policy makers to amend laws and regulations. Already the United Nations 2030 Sustainable Development Agenda (SD3) emphasizes wellness as a central component [56]. As witnessed in the four industrial revolutions, economies of scale will likely result in Connected Health technologies becoming affordable commodity products globally and with appropriate health policies, can contribute to sustainable healthcare for the future.
